# Nuchal cord and its implications

**DOI:** 10.1186/s40748-017-0068-7

**Published:** 2017-12-06

**Authors:** Morarji Peesay

**Affiliations:** Department of Pediatrics Division of Neonatal Perinatal Medicine, MedStar Georgetown University Hospital, 3800 Reservoir Rd, NW; Suite M3400, Washington, DC 20007 USA

**Keywords:** Nuchal cords, Tight Nuchal cord around the neck syndrome (tCAN syndrome), Partial prolonged asphyxia, Adult strangulation

## Abstract

Nuchal cord occurs when the umbilical cord becomes wrapped around the fetal neck 360 degrees. Nuchal cords occur in about 10–29% of fetuses and the incidence increases with advancing gestation age. Most are not associated with perinatal morbidity and mortality, but a few studies have shown that nuchal cord can affect the outcome of delivery with possible long-term effects on the infants. Nuchal cords are more likely to cause problems when the cord is tightly wrapped around the neck, with effects of a tight nuchal cord conceptually similar to strangulation. Umbilical cord compression due to tight nuchal cords may cause obstruction of blood flow in thin walled umbilical vein, while infant’s blood continues to be pumped out of the baby through the thicker walled umbilical arteries causing hypovolemia, acidosis and anemia. Some of these infants have physical features secondary to tight nuchal cords that are distinct from those seen with birth asphyxia. The purpose of this article is to review the pathophysiology of tight nuchal cords and explore gaps in knowledge and research areas.

## Background

In 1962, J. Selwyn Crawford [1] defined nuchal cord “360 degrees around the fetal neck.” Historical interest in nuchal cords has waxed and waned. In 1770, the first edition of the Encyclopedia Britannica (P.421), there were 20 pages written about Umbilical cord pathology including drawings of umbilical cord entanglement. In a book published in 1896, Gould [2] referred to a statement by Hippocrates (460 BC –ca. 370 BC) on nuchal cord as one of the dangers of the eighth month, stating that a nuchal cord persisting until the term, “will cause suffering to the mother and either perish or born difficulties to the fetus.” A chapter in Williams Obstetrics (16th Edition, 1980) states, “Coils (nuchal cords) occur in about 25% of cases and ordinarily do no harm, but occasionally they may be so tight that constriction of the umbilical vessels and consequent hypoxic result”. The implications of nuchal cords are controversial. Several studies have noted an association between nuchal cords and adverse perinatal outcome. In addition umbilical cord compression due to tight nuchal cords could be an incidental finding that is seldom associated with perinatal morbidity.

### Incidence

**Fig. 1:**
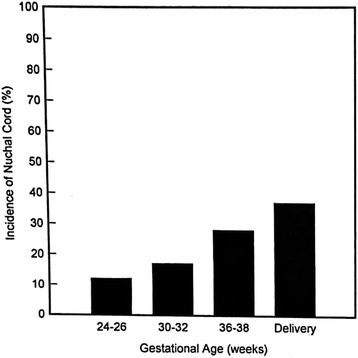
The incidence of nuchal cords at the four time points studied (Courtesy from Elsevier, with permission)

**Fig. 2:**
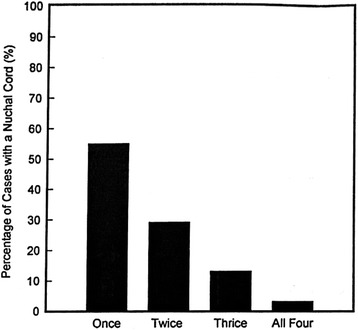
The frequency of occurrence in individual fetuses who had nuchal cord indentified during the four evaluations (Courtesy from Elsevier, with permission)

Larson JD et al. [3] reported that the overall incidence of nuchal cords was 6% at 20 wks GA and 29% at 42 weeks of gestation. Presence of two or more loops of nuchal cords is estimated to affect between 2.4% to 8.3% of all pregnancies. These entanglements may form early in gestation, although definitive evidence is lacking about exact time of its formation. Clapp, et al. [4] also showed increased incidence of nuchal cords with advancing gestational age irrespective of whether the entanglement involved single or multiple loops but without persistence (Figs. 1 and 2). This apparent inconsistency of increased incidence without persistence that Clapp noted was probably related to widely space ultrasonographic evaluations (3–6 weeks) and limited sampling times (5–10 min). In a large multicenter retrospective study, Henry, et al. [5] observed incidence of tight nuchal cord around by 6.6% and 21.6% loose nuchal cord. In his retrospective study Miser et al. [6] showed no influence of maternal age, race, parity on the incidence of nuchal cords but more common in male infants [6].

### Biomechanics of umbilical cord

There is limited literature on mechanics of umbilical cords. Ferguson, et al. [7] described bioengineering aspects of the umbilical cord emphasizing that “the umbilical cord is an extension of the fetal cardiovascular system with a great potential for use in studying changes within the fetal vascular tissues.” The constituent tissues of umbilical cord include outer layer of amnion and inner porous Wharton’s jelly, two umbilical arteries and one umbilical vein. The outer amnion layer seems to regulate fluid pressure within the umbilical cord. Wharton’s Jelly is a porous structure with poroelastic behavior and has proteoglycans and hyalauronic acid. It is supposed to protect against constriction due to fetal grasping, labor contractions, knots, kinks, or loops. On average, umbilical cords are 45–55 cm long, diameter of 1–2 cm and 11 helices, but diameters up to 3 cm and helices of as many as 380 per cord have been described [8]. Krakowiak, et al. [9] defines short cords as less than 40 cms and occur in approximately 6% of pregnancies. He notes that when short cord are around the neck, they are more likely to wrap tightly and are more often associated with decreased fetal movements, risk of cord compression, constriction and rupture. Presence of short cords seems to double and triple predictive values of low Apgar scores, low IQ values, neurologic abnormalities and stillbirths. Naeye, et al. [10] showed that long cords (defined as >70 cm length) have poor fetal outcome, higher fetal entanglements, true knots (sometimes multiple) and they are prone for torsion.

The biometrics patterns of uterine, fetal, umbilical cord lengths coupled with placental and amniotic fluid volumes could possibly be playing a role in formation of nuchal cords. Umbilical cords have elastic stretch up to 12.5% of its length [11]. Most cord ruptures happen within 12 in. of the fetus; 22% of the time ruptures occur at the placental end of umbilical cord. Studies have shown that traction forces of 8 lbs. usually separate the placenta from the uterus. Tensile strength of umbilical cord indicates that the average load required to break the cord is around 10–14 lbs. [11]. Majority of umbilical cords coil to the left in anticlockwise at ratio of 7 to 1 [12]. Mechanism of coiling includes intrinsic properties such as growth of vessels, differential blood flow within arteries and fetal movements. While exact causes for left twists are unknown, one of possible mechanisms seem to be that right umbilical artery is usually larger than left, which creates differential flow patterns resulting in left twist of umbilical cord. During embryonic development, physiological gut herniation occurs around 6–8 weeks of gestational age and subsequent reentry of herniated gut is usually anticlockwise and suspected to be another possible mechanism for common left twists of umbilical cords. Gupta et al. [13] defined hypocoiling, defined as <1 coil/5 cm and showed that it is associated with fetal distress, oligohydramnios, preterm delivery, intrauterine growth retardation, meconium stained amniotic fluid, fetal heart rate (FHR) alterations and low cord pH. Horn et al. [14], found association between hyper coiling (more than 1 coil/5 cm) and thinning with consecutive constriction of the umbilical vessels (thin cord syndrome; TCS) and intrauterine fetal death (IUFD). Noncoiled [15] umbilical cords are considered as risk factor for poor perinatal outcome and stillbirth.

### Pathophysiology

**Fig. 3:**
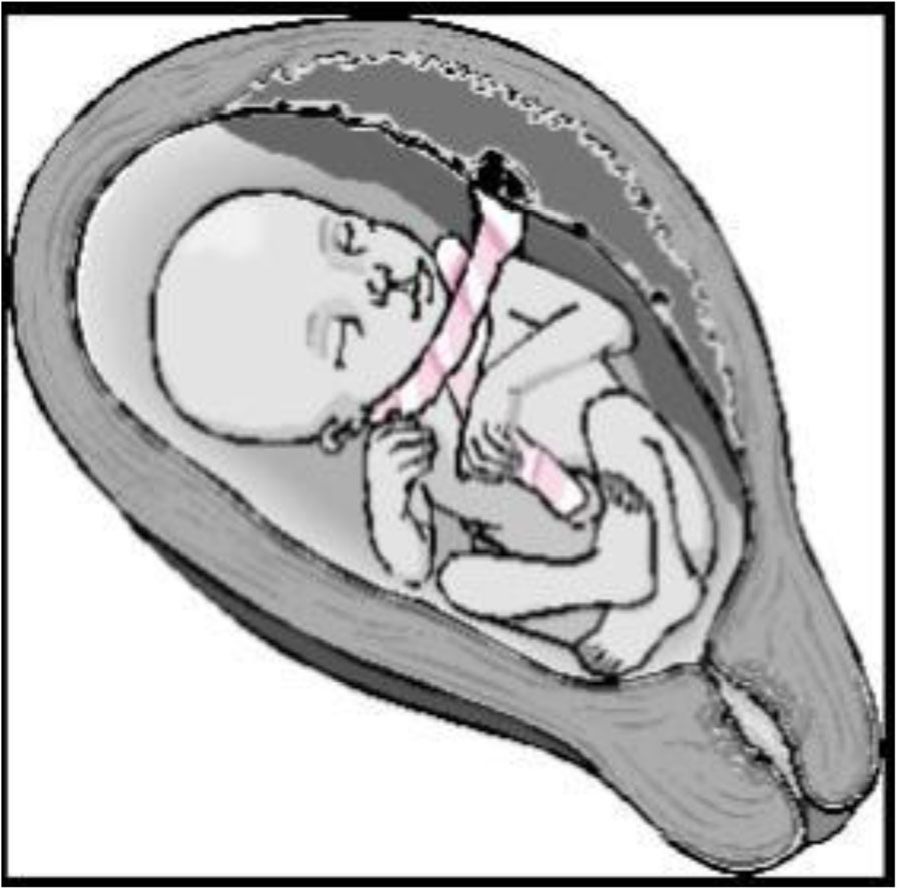
Nuchal Cord - Free sliding pattern: (Courtesy Philippe Jeanty et al., with permission)

**Fig. 4:**
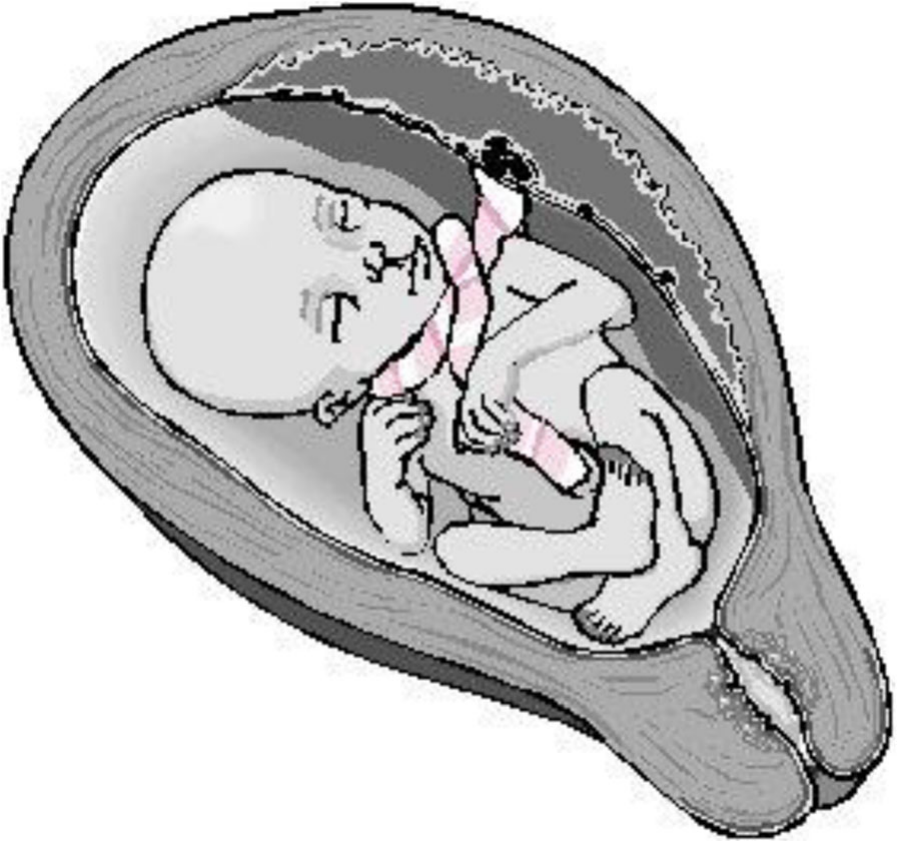
Nuchal Cord - Locked pattern (Courtesy from Philippe Jeanty et al., with permission)

**Fig. 5:**
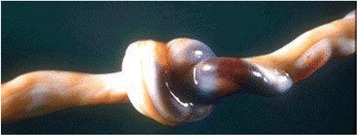
Umbilical Cord Knot. Notice the area of congestion to the right. The congested side is always the side between the knot and the placenta (Courtesy from University of Mo. Department of Pathology, with permission)

Nuchal cords can be single or multiple loops, and loose or tightly wrapped around the neck. Giacomello, et al. [16] recognized two types of nuchals: Type A- a freely sliding pattern which can undo itself (Fig. 3), and Type B - nuchal loop that encircles the neck in a locked pattern and cannot undo itself (Fig. 4). Very rarely a locked loop can be unlocked spontaneously by fetus that seems to be one of the mechanisms of true knot formation. Knots can be single or multiple (Fig. 5). If there is a nuchal cord at the onset of labor, it is very unlikely to correct itself. If there is no nuchal cord during prelabor, it is unlikely to occur during labor. Nuchal cords, especially when tightly wrapped, seem to have some similar physical features as those seen in strangulations. They include duskiness of face, facial petechiae, and conjunctival hemorrhage. A review article by Stapczynski et al. [17] on strangulations, describes presence of facial petechiae as a hallmark of jugular vein occlusion (lateral neck compression).When the jugular vein is occluded for ~15–30 s, facial petechiae seem to appear. Vagal collapse results from pressure to the carotid sinuses and increased parasympathetic tone. Other described findings of strangulation include retinal hemorrhages, hemotympanum and hyoid bone fractures (considered as pathognomonic of strangulation). The term compression asphyxia is used in adult Forensic Medicine, when strangulated victim torso is compressed by the perpetrator, suffers mechanically limiting expansion of the lungs interfering with breathing. As in normal delivery of newborns, nuchal cord babies’ chest is subjected to undue pressure during passage of vaginal canal and may suffer similar thoracic compression mechanically limiting expansion of the lungs. Long term effects of strangulations in adults include stroke and visual deficits. Nuchal cord may be considered worse than strangulation, since in nuchal cord, umbilical cord itself acts as a noose while carrying blood supply and the oxygen and umbilical vein being more vulnerable to collapse. Many of infants with nuchal cord pass meconium, which is probably an indication of vagal collapse. Based on pathophysiological findings mentioned above, author recommends developing grading system of tight nuchal cords as Grade 1: Conjunctival hemorrhage and petechiae. Grade 2: Duskiness of face, facial suffusion and pallor. Grade 3: Respiratory distress, stupor and hypotonia requiring resuscitation.

### Pathology

**Fig. 6:**
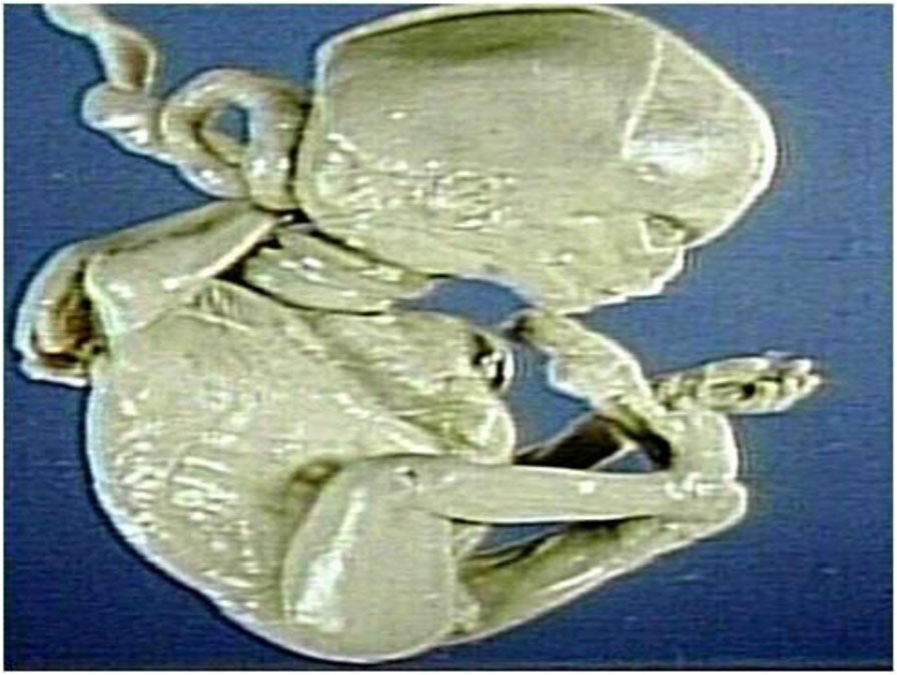
Pathology specimen of fetus with multiple nuchal cords (Courtesy from Peter Anderson PEIR Digital Library, with permission)

Pathology specimens speak volumes about the rare lethal effects of tight nuchal cords (Fig. 6) leading to stillbirth. Baergen, et al. [18] found that as much as 20% of stillbirths at autopsy are due to fatal compromise of umbilical circulation. Parast, et al., [19] for the first time established evidence of placental histologic criteria for umbilical blood flow restriction (cord accident) in unexplained stillborns. Placental slides from 62 cases of third trimester unexplained stillbirths reviewed to define criteria and estimate the frequency of cord accident as cause of stillbirth. By correlating clinical and autopsy information with placental gross and histologic findings from a series of index cases with a strong presumptive evidence of cord accident, minimal histologic criteria for cord accident were established. “Minimal histologic criteria” suggestive of cord accident were defined as vascular ectasia and thrombosis within the umbilical cord, chorionic plate, and/or stem villi. Of 27 stillbirth cases (out of 62 cases reviewed) who had a cause of death determined to be other than cord accident only 3(11%) met all histologic criteria for cord accident(specificity of 89%). In contrast of remaining 25 stillbirth cases with an unknown cause of death, a significantly larger subset (13 cases or 52%) met the criteria for cord accident (*p* = 0.0038). All stillbirth fetuses and placentas that met the histologic criteria for cord accidents exhibited marked normoblastemia, reflecting possible subacute to chronic hypoxic stress prior to demise. Although normoblastemia is by no means specific for cord accident it is a constant finding in nonacute fatal restriction of umbilical blood flow (cord accident).

### Clinical features

**Fig. 7:**
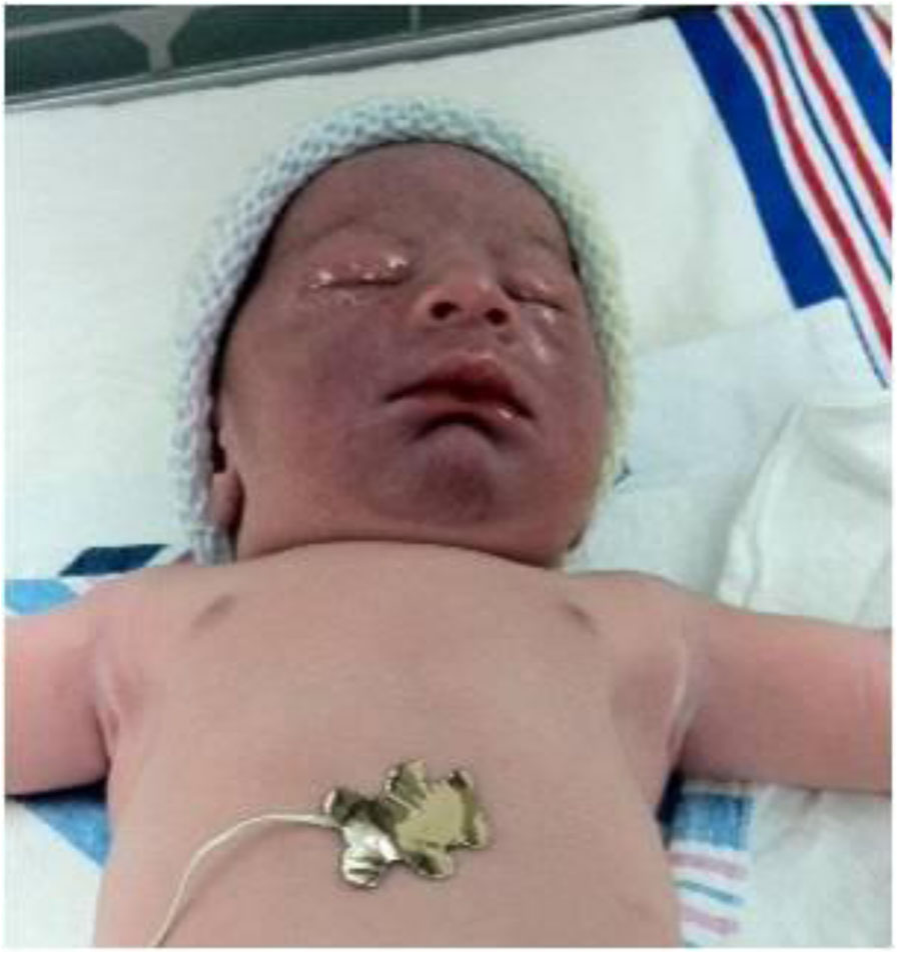
Facial Duskiness due to tight Nuchal cord

**Fig. 8:**
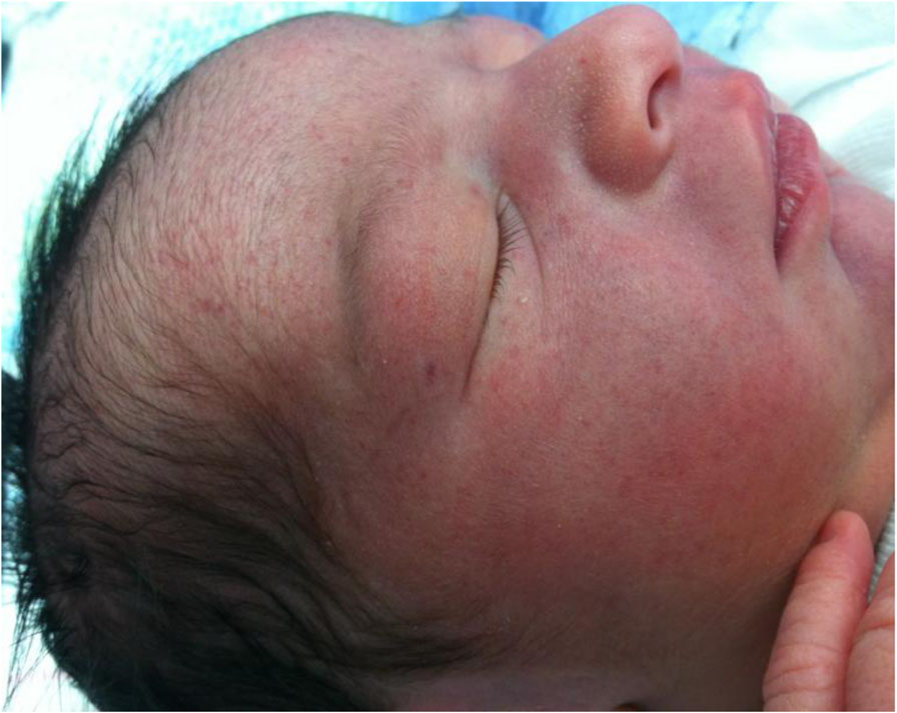
Petechiae on face due to tight nuchal cord

**Fig. 9:**
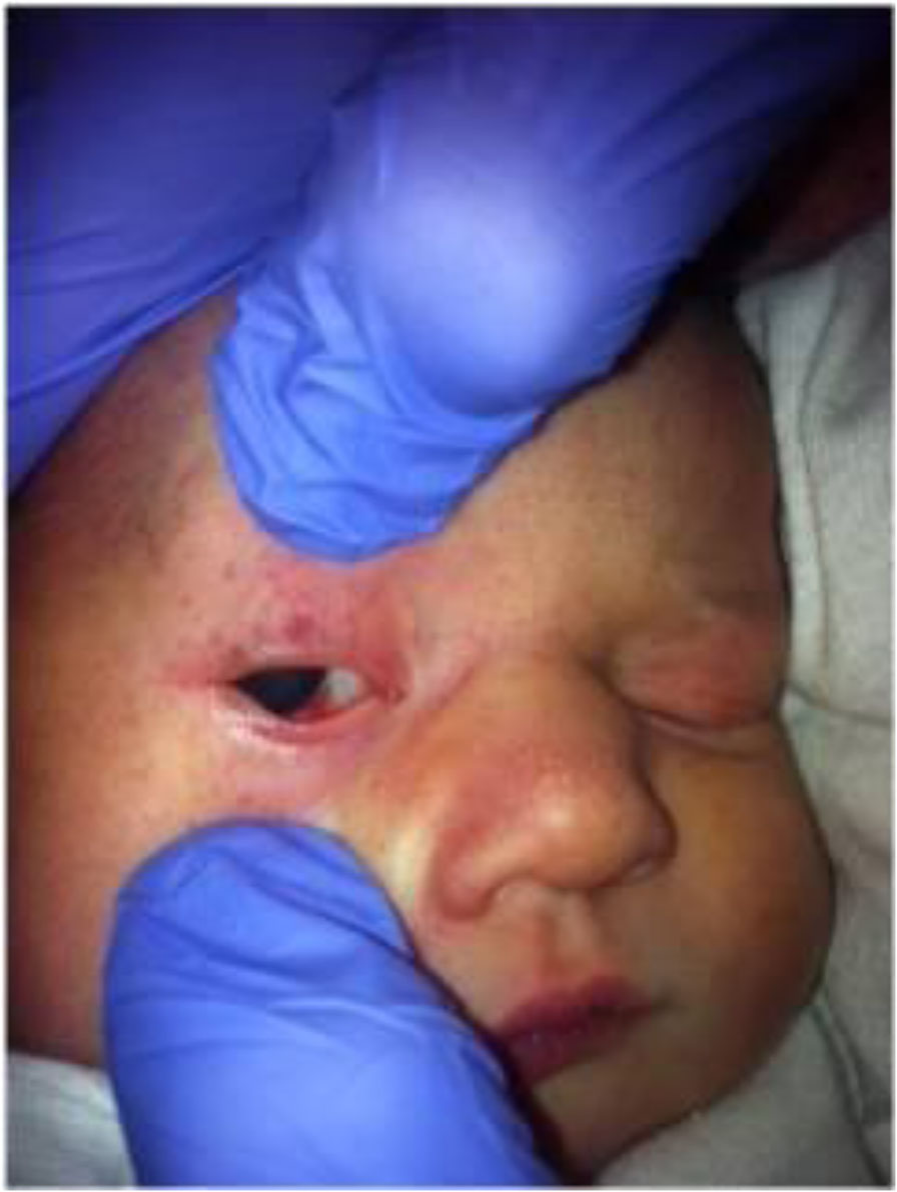
Facial Petechiae and Subconjuntival hemorrhage due to tight Nuchal cord

**Fig. 10:**
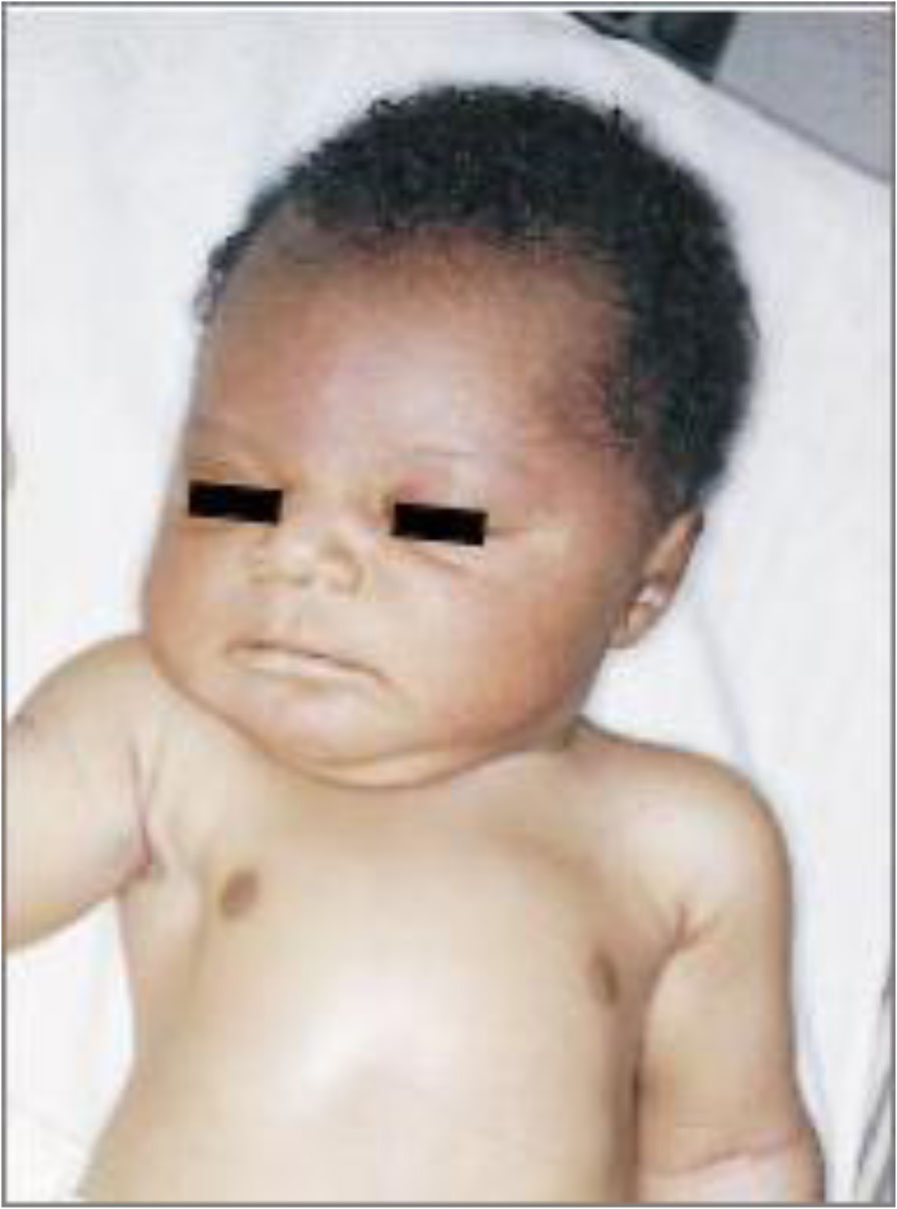
Suffusion of the face and head due to tight nuchal cord. (From Arnold J. Rudolph, M.D. Atlas of the Newborn, B.C Decker Inc., with permission)

**Fig. 11:**
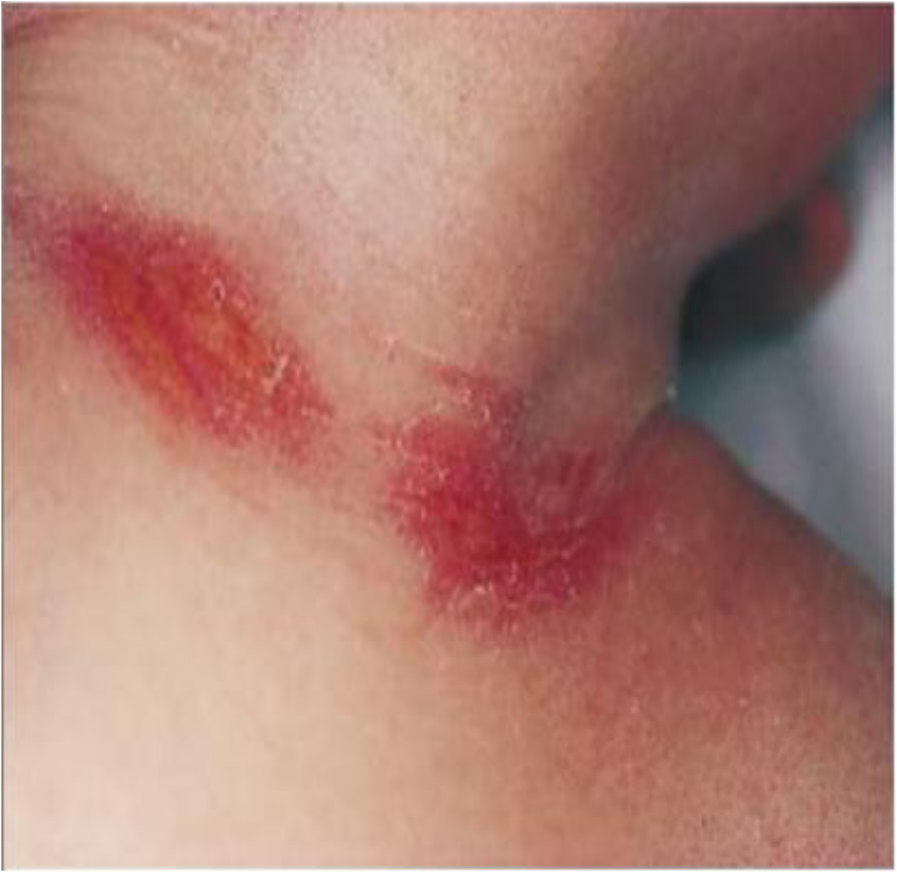
Nuchal Cord with Skin abrasion of neck - Cord Around the Neck times three. (From Arnold J. Rudolph, M.D. Atlas of the Newborn, B.C Decker Inc., with permission)

Infants with a tight nuchal cord may develop signs and symptoms such as hypovolemia, hypotension, decreased perfusion and mild respiratory distress. Other findings occasionally noted may include facial duskiness (Fig. 7), facial petechiae (Fig. 8), subconjuntival hemorrhage (Fig. 9), facial suffusion (Fig. 10), or skin abrasion around the neck (Fig. 11) due to tight nuchal cords. Rarely, infants become neurologically depressed or hypotonic with depressed neonatal reflexes. Based on cardio-respiratory and neurological signs and symptoms associated with a tight nuchal cord, one can group the common findings together into a possible syndrome called tight Cord Around the Neck syndrome, or ‘tCAN syndrome. We propose this term as “a cluster of cardio-respiratory and neurological signs and symptoms associated with unique physical features that occurs secondary to tight cord-round-the-neck” [20]. Rarely, there can be significant blood loss, acidosis, and anemia. A few small studies and case reports show nuchal cord as a cause of neonatal anemia. Shepherd, et al. [21] reported anemia in 5 out of 27 (19%) infants with tight nuchal cord. Three infants had hypotension requiring blood transfusion. Asymptomatic anemia as a complication of loose nuchal cords was also described. Vanhaesebroudk, et al. [22] published a case report of two neonates with acute hypovolemic shock requiring blood transfusion, low APGAR scores and umbilical arterial acidosis after tight nuchal cord. The author also noted from personal experience that if the finding in babies with a history of a loose nuchal cord include one or all of the findings such as facial petechiae and a dusky face, subconjuntival hemorrhage and low umbilical arterial cord pH, it is probably a tight nuchal cord at variable period of time prior to delivery. It is also authors’ personal observation that infants with hypermagnesemia due to maternal magnesium sulfate treatment with history of tight nuchal cord show increased risk of apneas and desaturations, possibly due to their combined effect on central nervous system. As of now, there is no scientific evidence supporting these personal experiences.

### Diagnosis

**Fig. 12:**
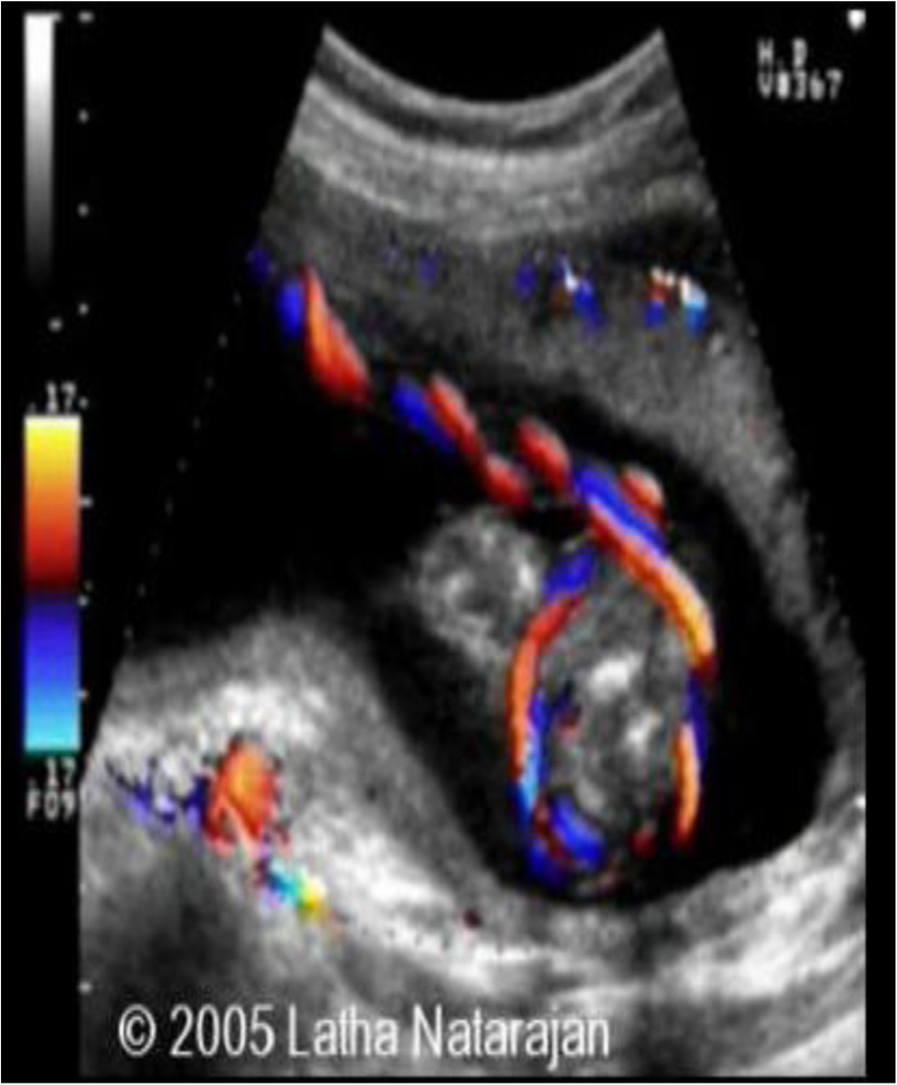
Fetal Ultra sound color doppler scan of Nuchal Cord (Courtesy from Dr. Latha Natarajan, with permission)

**Fig. 13:**
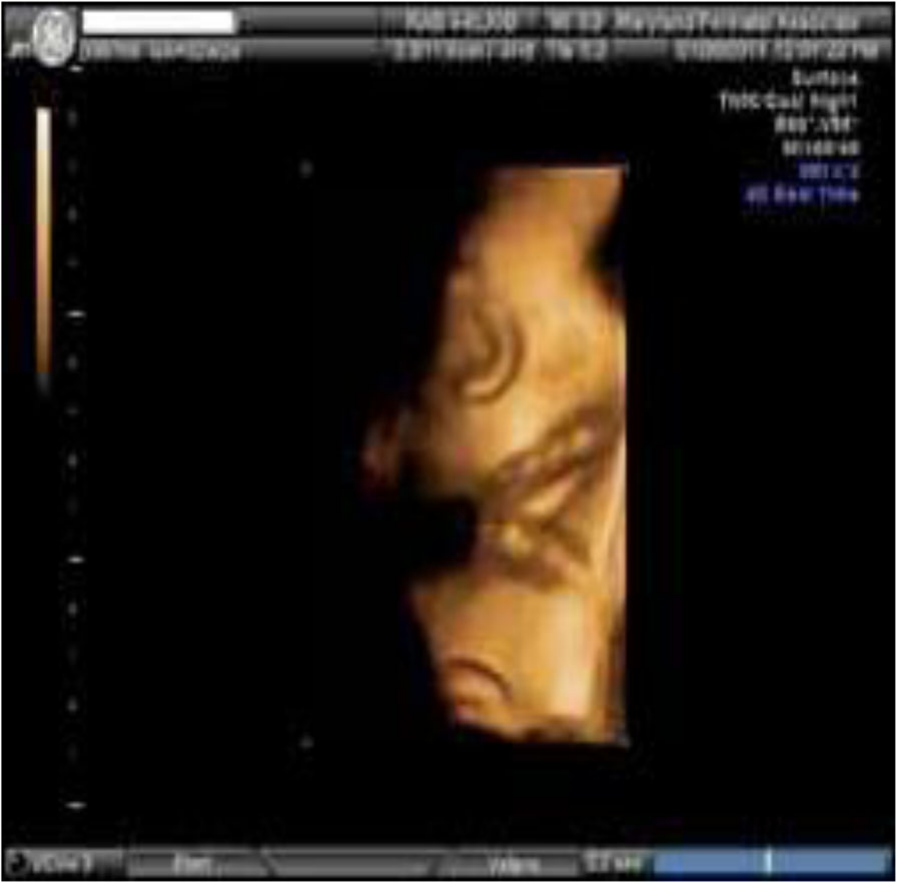
Fetal Ultra Sound scan - 3D image of double nuchal cord (Courtesy from Maryland Perinatal Associates, with permission)

One of the causes of variable decelerations in antenatal fetal heart rate monitoring is nuchal cord. Nuchal cords can be diagnosed antenatally by ultrasonography (Figs. 12 and 13). Jauniaux, et al. [23] showed ultrasonography to be the gold the standard when combined with color Doppler imaging. This seems to correctly identify 72% of single and 94% of multiple nuchal cords, with greatest sensitivity after 36 weeks (93% vs. 67%). Ultrasonographers can look for a “divot” sign on high-resolution ultrasound [24], a circular indentation of the fetal nuchal skin; but care should be exercised not to confuse this finding with posterior cystic masses, folds of skin, or amniotic fluid pockets. Prior to delivery, obstetricians can assess presence of nuchal cords clinically by a test that involves transabdominal manual compression of the fetal neck. If compression of fetal neck elicits fetal heart rate decelerations (FHR), the test is considered positive. This indicates impending risk of cord compression and is an indication for close FHR monitoring. Mendez-Bauer, et al. [25] showed in his study that this noninvasive test has sensitivity of 82.3% and specificity 89.1%. These results were statistically significant in both late pregnancy and labor. A positive test is an indication for close electronic fetal monitoring, particularly during labor. Study concluded that routine use of this test can contribute to decreasing perinatal morbidity and mortality by diminishing the impact of cord problems. One can also diagnose presence of nuchal cords noninvasively prior to delivery, by using vibroacoustic stimulation (electronic artificial larynx) which at particular frequencies of vibrations seem to elicit fetal heart rate decelerations. Anyaegbunam, et al. [26] used vibroacoustic stimulation during second stage of labor eliciting accelerations, decelerations, and acceleration followed by decelerations. The incidence of nuchal cord was significantly higher for the group with a response pattern of fetal heart rate acceleration followed by deceleration than for the acceleration or no-response groups (39.2% versus 10.5% versus 11.1%; *P* < .05).

### Fetal hypoxia

Nuchal cords can cause cord compression, leading to obstruction of blood flow in the thin walled umbilical vein, while blood continues to be pumped out through the thicker walled umbilical arteries causing hypovolemia, hypotension and fetal hypoxia [22]. There are only few studies that have focused on tight nuchals causing fetal hypoxia. Xu, et al. [27] investigated the value of middle cerebral artery (MCA) and umbilical artery (UA) resistance index in predicting fetal hypoxia in fetuses with umbilical cord around the neck in late pregnancy. Included in this study are measurements of MCA and UA pulsatility index (PI), resistance index (RI), peak systolic and diastolic (S/D) ratios, and resistance index ratios (RIR). RIR showed no significant difference between the no nuchal cord and the 1-loop nuchal cord group (*P* > 0.05). However, significant differences were noted between the no nuchal cords group and the multiple nuchals group (*P* < 0.01), and between the ≥2-loops nuchal cord group and the 1-loop nuchal cord group (*P* < 0.05). One of the conclusions of study is that MCA/UA RIR compared to PI, RI, S/D ratios is a better indicator for predicting fetal hypoxia in nuchal cord infants. Hashimoto, et al. [28] investigated evidence of fetal hypoxia by studying levels of erythropoietin (EPO) in umbilical cord blood and amniotic fluid. Tissue hypoxia is the major stimulus of erythropoietin (EPO) synthesis in fetuses and adults. Previous studies have shown that EPO does not cross the placenta [29], and that plasma EPO levels correlate with the intensity of hypoxia [30]. Cord blood EPO levels increase in acute fetal hypoxia, and amniotic fluid (AF) EPO levels increase in chronic fetal hypoxia. Hashimoto, et al., showed that in the absence of ante/intra partum complications, higher levels of AF-EPO at birth were more predictive of nuchal cord whereas cord blood EPO levels were not found to have any correlation with nuchal cord. Tightness of the nuchal cord did not affect AF or cord blood EPO concentrations. This study concluded that nuchal cords may not significantly increase the risk of acute or labor-associated fetal hypoxia, but are an independent risk factor of mild, chronic, prelabor fetal hypoxia.

### Prolonged partial asphyxia evidence

**Fig. 14:**
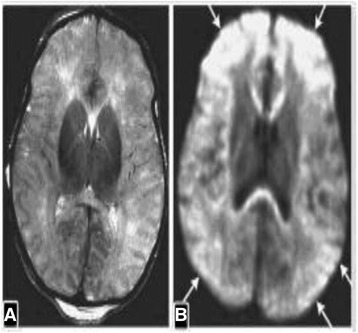
MRI scans of severe Partial Prolonged Asphyxia**:** A 4-day-old infant owing to tight cord around neck. A) Axial T2- weighted imaging shows abnormal cortical and subcortical bilateral cerebral increased T2 signal intensity. B) Axial diffusion weighted imaging shows extensive gray and white matter injury (arrows) of both cerebral hemispheres – From KN V: Cerebral Palsy and Early Stimulation. New Delhi, India: Jaypee Brothers. Medical Publishers (P) Ltd., 2014, with permission

Severe prolonged partial asphyxia could be one of the rare complications of tight nuchal cords [31]. Causes of prolonged partial asphyxia (PPA) include nuchal cord, placental failure and tetanic uterine contractions. In prolonged partial asphyxia there is gradual reduction in blood flow and oxygen over several hours to the brain, probably due to occlusion of intramyometrial segment of uterine and or umbilical artery, while cardiac pumping is generally preserved. Brain injury in PPA primarily affects the cerebral cortex and is associated with severe brain swelling in the watershed areas (especially in the parasagittal region). The vascular watershed zones (anterior–middle cerebral artery and posterior–middle cerebral artery) are involved, thereby affecting white matter (Fig. 14). PPA could be one of the rare complications of tight nuchal cords.

### Nuchal cords and birth asphyxia

One of several attempts to distinguish and delineate the effects of nuchal cords from birth asphyxia was done by Martin, et al. [32] in a study on umbilical cord gas acidosis comparing newborns with and without nuchal cords that had normal APGAR scores. They found that while umbilical venous (UV) pH, pCO2, and oxygen content were not statistically different between the two groups, infants with nuchal cord had significantly lower pH, lower oxygen content, and higher PCO2 levels in their arterial cord gases. The Veno-Arterial (VA) differences in pH and PCO2 of the infants with nuchal cord were greater than that of the infants with no nuchal cords. The study concurred with other studies that Apgar score is not a sensitive indicator of acid-base changes in nuchal cord infants. Umbilical venous blood gases can be misleading in these infants, and therefore umbilical arterial blood must be sampled to detect hypercapnia and diminished oxygen content resulting from a nuchal cord. A significant umbilical arterial acidosis can occur in nuchal cord infants even in the setting of normal or near normal APGAR scores. Belai, et al. [33] showed that APGAR scores correlate very weakly with cord pH when the pH is greater than 7.0. We speculate that the physical features of tCAN syndrome may distinguish perinatal depression due to nuchal cords from other causes of birth asphyxia. Miriam Martinez-Biarge et al. [34] in 2013 in their retrospective analysis to determine whether antepartum factors alone, intrapartum factors alone, or both in combinations, are associated with term neonatal hypoxic-ischemic encephalopathy (HIE). A total of 405 infants >35 weeks’ gestation with early encephalopathy, were compared with 239 neurologically normal infants newborns. All cases met criteria for perinatal asphyxia, had neuroimaging findings consistent with acute hypoxia-ischemia, and had no evidence for a non–hypoxic-ischemic cause of their encephalopathy. On logistic regression analysis only 1 antepartum factor (gestation > 41 weeks) and 7 intrapartum factors (prolonged membrane rupture, abnormal cardiotocography, thick meconium, sentinel event, shoulder dystocia, tight nuchal cord, failed vacuum) remained independently associated with HIE (area under the curve 0.88; confidence interval 0.85–0.91; P, .001). Tight nuchal cord was noted in 45/396 cases and 15/237 in control group with odds ratio of 1.89 (1.03–3.5). Their results do not support HIE is attributable to antepartum factors alone, but they strongly point to the intrapartum period as the necessary factor in the development of this condition. It is our speculation that the established terminology ‘Compression Asphyxia’ in forensic medicine [35] could be applied to these infants with tight nuchal cords because of the similarities in their patho-physiological mechanisms and clinical findings. This may help in distinguishing birth asphyxia from infant with the effects of a tight nuchal cord.

### Prognosis

Several animal studies have been conducted in order to simulate the effects of nuchal cords. Rocha, et al. [36] examined the effect of intermittent umbilical cord occlusion during brain development in preterm and near-term ovine fetuses. The purpose of this study was to determine the immunoreactivity of selected structural proteins in the preterm and near-term ovine fetal brain and the response to intermittent umbilical cord occlusion as a measure of altered cellular growth. The intermediate filament proteins nestin, vimentin, and glial fibrillary acidic protein was used as markers for astroglial maturation and astrogliosis, and myelin basic protein as a marker for oligodendrocytes and myelin formation. Fetal sheep (control and experimental groups at 0.75 and 0.90 of gestation) were studied over 4 days; umbilical cord occlusion was performed in the experimental group by complete inflation of an occluder cuff for 90 s every 30 min for 3 to 5 h each day. Animals were then euthanized, and the fetal brain was perfusion fixed and processed for immunohistologic examination of the gray and white matter. In both preterm and near-term animal groups, umbilical cord occlusion caused a large decline in arterial Po2 (to approximately 7 mmHg), a modest decline in pH (to approximately 7.30), and a modest rise in PCo2 (to approximately 61 mmHg; all *P* < .01), with a return to control values after the occluder release and no cumulative acidosis over each day of study. Vimentin and glial fibrillary acidic protein immunoreactivity showed reciprocal changes, with vimentin decreased and glial fibrillary acidic protein increased in both the gray and white matter of the control group from 0.75 to 0.90 of gestation, which can be attributed to the transition of radial glia into mature astrocytes. Myelin basic protein immunoreactivity increased approximately 3-fold in the white matter of the control group with advancing gestation, which likely reflected active oligodendrocyte differentiation and increased myelination at this time of development. Intermittent umbilical cord occlusion over 4 days resulted in an approximately 60% decrease in nestin, vimentin, and glial fibrillary acidic protein immunoreactivity, which was qualitatively similar for both the gray and white matter and likely indicative of altered protein synthesis and/or degradation, but only in the preterm group and with no change in myelin basic protein immunoreactivity. The results showed that intermittent umbilical cord occlusion as studied with minimal evidence for necrotic cell injury appears capable of altering selected protein synthesis/degradation, more so in younger animals when protein turnover is higher, which might then impact on the brain’s development. Due to its common occurrence, mostly benign nature, multifactorial causality, and lack of clear guidance towards management, the long-term neurological injury secondary to nuchal cords has been debated. Some studies published on neonatal outcome include fetal demise and neurological impairments, while the vast majority of studies have shown no long term neurodevelopmental impairment. Yet a very small, but significant, percentage of infants with tight nuchal cords seem to face devastating effects as evidenced in pathophysiological features in tCAN syndrome. In a retrospective study of normal birth weight infants with spastic cerebral palsy, Nelson, et al. [37] showed an association between tight nuchal cords and quadriplegia. In this observational study, none of the infants with diplegia or hemiplegia had potentially asphyxiating conditions. In their study on asphyxia-related risk factors and their timing in spastic cerebral palsy (CP), Neilson, et al. [38] noted a threefold increase in risk of spastic CP in infants with tight nuchal cords. Clapp, et al. [39] performed a prospective study examining neurodevelopmental performance at 1 year of age in infants who had nuchal cords at delivery. Infants of 190 women with clinically normal antenatal courses were evaluated within 1 month of their first birthday by a blinded observer using the Bayley Scales of Infant Development. The cases were grouped based on the presence of a symptomatic nuchal cord (defined as an abnormal fetal heart rate patterns or meconium) during labor and fetuses with symptomatic nuchal cord presentations with one or more additional anatomic findings suggesting increased risk were identified as a symptomatic-complicated subgroup. The additional anatomic findings included the following: nuchal cords that were identified initially as an incidental ultrasound finding 4 or more weeks before labor, which presumably persisted until the time of birth, multiple loops around the neck at birth, an associated true knot or an excessively tight nuchal cord at the time of birth. At 1 year of age scores on both Bayley scales were slightly but significantly (*P* < .01) lower in the offspring delivered with a symptomatic nuchal cord. The mental index was 116 +/− 9 versus 120 +/− 7, and the psychomotor index was 101 +/− 11 versus 107 +/− 9. These differences were accentuated (*P* = .09) when the symptomatic cases complicated by extreme tightness, multiple loops, or antenatal detection were compared to symptomatic cases without these additional complications (overall index 110 +/− 8 versus 105 +/− 10). The differences are not statistically significant, suggesting that this may be largely due to the performance of the offspring born with a symptomatic presentation that was complicated also by additional anatomic findings. There were no between group differences in multiple potential confounding obstetric or demographic variables. These data suggest that symptomatic nuchal cords, which are identified before labor as being extremely tight or having multiple loops, may be associated with a subclinical deficit in neurodevelopmental performance at 1 year of age. There was marked reduction in birth weights seen in about ~25% of fetuses with persistent nuchal cord associated with low middle cerebral artery systolic and diastolic ratios, this is suggestive of a chronic hypoxic state. In contrast, Greenwood, et al. [40] showed that reported associations of nuchal cord with cerebral palsy (CP) were influenced by recording bias. There were 68 cases with cerebral palsy and 157 controls (singleton term infants matched for gestational age and hospital of birth). CP was associated with tight nuchal cord overall (OR = 2.8, 95% CI 1.1–6.8). Where cord around the neck is recorded at the discretion of the accoucheur (37 cases, 97 controls), there was an association between tight nuchal cord and CP (OR = 5.4, 95% CI 1.4–20.4) and, apparent association between nuchal cords and Apgar score < 7 at 1 min (OR = 16.9, 95% CI1.4–456.3). In the hospital where records included a tick box for nuchal cord (31 cases, 60 controls), an association between CP and tight nuchal cord could not be demonstrated (OR = 1.4, 95% CI 0.4–4.9) nor was there an apparent association between nuchal cord and Apgar score < 7 at 1 min (OR = 2.6, 95% CI 0.4–15.9) in controls. Thus showing that an association of CP with nuchal cord is not evident where documentation is systematic. In a retrospective study, Sheiner, et al. [41] concurs with nuchal cord not being associated with adverse perinatal outcomes. In a large multicenter retrospective study, Henry, et al. [5] observed no difference in outcomes following tight nuchal cords. Using electronic data of 219,337 live births over 6-year period (2005 to 2010) in a multihospital healthcare system in the Western United States, they reported an incidence of tight nuchal cord of 6.6% and an incidence of loose nuchal cords of 21.1%. Neonates with tight nuchal cords were slightly more likely to be admitted to a NICU (6.6% vs. 5.9% admission rate, p 0.000). In this study, no difference in outcome was seen in very low birth weight (VLBW) infants with tight nuchal cords. In a retrospective analysis of a perinatal database including 11,748 vaginal deliveries, Schaffer, et al. [42] noted significantly more male than female infants in the nuchal cord group, 52.9% in all nuchal cords (*p* < 0.5) and 54.5% in multiple nuchal cords (p < 0.5). The rates of vaginal operative deliveries or deliveries by cesarean were not significantly increased in any nuchal cord group in term and post term deliveries. Schaffer also noted that there was significantly lower birth weights were found in all nuchal cord groups suggesting potentially prolonged chronic mild fetal hypoxia caused by nuchal cord. This effect was especially pronounced in multiple cords, with a decrease of 93 g in term and 180 g in posterm multiple nuchal cords (*p* < 0.05). Zhang, et al. [43] used a questionnaire to examine possible prenatal and perinatal risk factors for autism in a case control study in Hans Province, China. One hundred and ninety children with autism were recruited from public special education schools, with controls from regular public schools in the same region. Nuchal cord occurred with a higher frequency among children with autism (23.2%) than among the controls (6.3%) (*p* = 0.002).

### Obstetrical management

The obstetrical challenge of the clinical management of nuchal cords depends upon number of involved nuchal loops, the amniotic fluid index, the gestational age, and the fetal growth, among other factors. Induction of labor considered as independent risk factor for nuchal cords. A prolonged persistent nuchal cord with poor fetal growth deserves close monitoring and delivery as appropriate. Some obstetricians opt to deliver early when multiple nuchal cord loops are noted on fetal scans. Presence of variable decelerations during fetal heart rate monitoring is indicative of possible presence of nuchal cord.

## Conclusions

Nuchal cords are a potential cause for perinatal distress and a rarely significant risk factor for long-term neurodevelopmental consequences in the developing fetus. A renewed interest is needed in diagnosing nuchal cords by judicious use of ultrasound with color Doppler, MCA and UA resistance index ratios, amniotic EPO levels, and possibly the use of manual abdomen compression or vibroacoustic stimulation tests. Examining placenta and cord pathology in cases of tight nuchal cords could also be informative. We speculate that ‘Compressional Asphyxia’ would be more appropriate terminology for neonates who are asphyxiated secondary to tight nuchal cords. Prospective analysis is needed by neonatologists to report the signs of tight nuchal cord as described in tCAN syndrome. Close follow-up in developmental clinics would help in predicting the outcomes. Studies exploring possible role of fundoscopy, otoscopy, and EEG may expand and help fill the knowledge gaps. Better understanding of bioengineering aspect of umbilical cord especially on mechanism of nuchal cord formation may shed light on management. There needs to be a focus on neuropathology to examine stillborn fetus brains histologic findings that might be unique to tight nuchals. Obstetricians may look for placental histological changes secondary to tCANs in infants born alive. Further study by pathologist on stillborn with tCANs may include fundoscopy for retinal hemorrhages, otoscopy to rule out hemotympanum, and X-ray of hyoid bone to rule out fractures. This would help clarify possible lethal aspects of tCANs.

The enormous anxieties mothers go through when their fetuses have locked pattern of nuchal cords continue to challenge obstetricians. From the perspective of neonatologists, who see evidence of tight nuchals as in tCAN syndrome and pathologists who witness during autopsy, tight nuchal cord as a cause stillbirth, nuchal cords do not seem to be benign.

## Abbreviations

(S/D) ratios: Peak Systolic and Diastolic ratios
AF: Amniotic fluid
CP: Cerebral Palsy
EPO: Erythropoietin
FHR: Fetal Heart Rate
FTV: Fetal Thrombotic Vasculopathy
IUFD: Intra Uterine Fetal Death
MCA: Middle Cerebral Artery
PI: Pulsatility index
PPA: Prolonged Partial Asphyxia
RI: Resistance index
RIR: Resistance Index Ratios
tCAN: tight Cord Around the Neck syndrome
TCS: Thin Cord Syndrome
UA: Umbilical artery
UV: Umbilical Venous
VA: Veno-Arterial
VLBW: Very Low Birth Weight
